# Precision Nutrition in Chronic Inflammation

**DOI:** 10.3389/fimmu.2020.587895

**Published:** 2020-11-23

**Authors:** Tobias J. Demetrowitsch, Kristina Schlicht, Carina Knappe, Johannes Zimmermann, Julia Jensen-Kroll, Alina Pisarevskaja, Fynn Brix, Juliane Brandes, Corinna Geisler, Georgios Marinos, Felix Sommer, Dominik M. Schulte, Christoph Kaleta, Vibeke Andersen, Matthias Laudes, Karin Schwarz, Silvio Waschina

**Affiliations:** ^1^ Division of Food Technology, Institute of Human Nutrition and Food Science, Kiel University, Kiel, Germany; ^2^ Division of Endocrinology, Diabetes and Clinical Nutrition, Department of Medicine 1, Kiel University, Kiel, Germany; ^3^ Research Group Medical Systems Biology, Institute of Experimental Medicine, Kiel University, Kiel, Germany; ^4^ Division of Nutriinformatics, Institute of Human Nutrition and Food Science, Kiel University, Kiel, Germany; ^5^ Institute of Clinical Molecular Biology (IKMB), Kiel University, Kiel, Germany; ^6^ Institute of Regional Research, University of Southern Denmark, Odense, Denmark; ^7^ Institute of Molecular Medicine, University of Southern Denmark, Odense, Denmark; ^8^ Focused Research Unit for Molecular Diagnostic and Clinical Research, University Hospital of Southern Denmark, Aabenraa, Denmark

**Keywords:** nutrition, personalized medicine, inflammation, dietary intervention, disease prevention, microbiota, precision nutrition, chronic diseases

## Abstract

The molecular foundation of chronic inflammatory diseases (CIDs) can differ markedly between individuals. As our understanding of the biochemical mechanisms underlying individual disease manifestations and progressions expands, new strategies to adjust treatments to the patient’s characteristics will continue to profoundly transform clinical practice. Nutrition has long been recognized as an important determinant of inflammatory disease phenotypes and treatment response. Yet empirical work demonstrating the therapeutic effectiveness of patient-tailored nutrition remains scarce. This is mainly due to the challenges presented by long-term effects of nutrition, variations in inter-individual gastrointestinal microbiota, the multiplicity of human metabolic pathways potentially affected by food ingredients, nutrition behavior, and the complexity of food composition. Historically, these challenges have been addressed in both human studies and experimental model laboratory studies primarily by using individual nutrition data collection in tandem with large-scale biomolecular data acquisition (e.g. genomics, metabolomics, etc.). This review highlights recent findings in the field of precision nutrition and their potential implications for the development of personalized treatment strategies for CIDs. It emphasizes the importance of computational approaches to integrate nutritional information into multi-omics data analysis and to predict which molecular mechanisms may explain how nutrients intersect with disease pathways. We conclude that recent findings point towards the unexhausted potential of nutrition as part of personalized medicine in chronic inflammation.

## Introduction

Over the past several decades, increased incidence rates of diseases associated with chronic inflammation, including inflammatory bowel disease (IBD), diabetes, and asthma have been observed in countries experiencing industrial and urban growth (1–3). While the causes of this incidence surge are still highly debated in biology and medicine communities, there is increasing epidemiological evidence that the rise of chronic inflammatory diseases (CIDs) can be attributed to nutritional changes (4–7). A dietary basis for CIDs is further supported by the fact that they frequently involve physiological changes in the gastrointestinal tract including alterations in gut microbiota composition and metabolism (8–11). In this context, a number of studies have identified molecular mechanisms by which dietary components can interact with immunological pathways either directly (12, 13) or indirectly, *via* modulation of the gut microbiota (14, 15).

Patients with the same CID can differ markedly in their precise disease manifestation with respect to inflammation relapse, remission, and response to therapy (16, 17). Studies using clinical cohorts have revealed several molecular features that are associated with disease heterogeneity. These include genetic (18, 19) microbial (20), and metabolic factors (10, 21). The appreciation of the wide range of individual factors influencing the pathology of CIDs intensified research endeavours to further tailor treatment strategies to the patient’s molecular characteristics (22, 23).

Given the multitude of molecular mechanisms by which nutrition can intersect with immunological pathways, microbiome dynamics, and human metabolism, nutrition therapy has been recognized as integral to the development of novel personalized CID prevention and disease management strategies (24). Additionally, nutrition has vast potential to contribute to personalized medicine in two ways: first, the patient’s nutritional status and dietary intake information can be used to inform new prescriptive biomarkers, i.e. biomarkers that can predict the patient’s response to potential treatment strategies (25). Second, nutritional interventions display promise for patient-centered treatments of CIDs.

**Figure 1:**
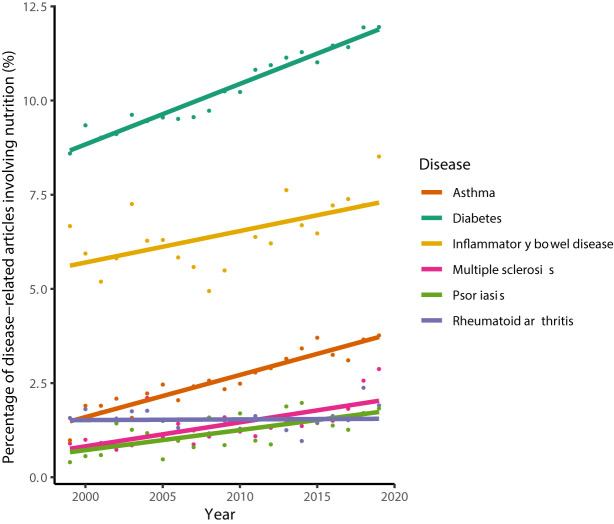
Percentage of CID-related articles, whose titles or abstracts involve the terms “nutrition' or “diet'. The search was performed on July 6th 2020 using PubMed^®^ and limited to the years 1999–2019. Lines denote the results from linear regression analysis. Percentages increased significantly for all diseases (Pearson’s product-moment correlation, *P* < 0.005) except for rheumatoid arthritis (*P* = 0.87).

Those ideas are mirrored in an increasing number of CID-related articles in the scientific literature that also involve aspects of nutrition and diet (**Figure 1**). Yet, empirical studies reporting clear evidence of the effectiveness of using nutrition-derived biomarkers and nutritional interventions in CID therapies are rare, thereby limiting nutrition data-assisted decision making and dietary interventions in clinical practice of personalized medicine. For instance, a recent systematic review combined with an expert survey to derive guidelines for clinical nutrition management in IBD yielded only 7 evidence-based dietary/nutrition recommendations that relate the patient’s individual characteristics, e.g. age, current and previous treatments, and nutritional status (26).

The large discrepancy between the anticipated role and actual application of nutrition in personalized CID management is largely due to the intrinsic biochemical complexity of nutrition, including its long-term effects and interaction with various environmental factors (27, 28). However, recent studies have started to address this issue by investigating the impact of nutrients on host organisms alongside the molecular interactions between nutrients, microorganisms, drugs, and host genetics (29). In this review, we highlight recent developments at the interface between nutrition and precision medicine in chronic inflammation. In addition, key challenges in the field are discussed and potential solutions proposed.

## Nutrition and Prescriptive Biomarkers

Nutrition is an important determinant of CID patient heterogeneity (30). Thus, specific information about an individual’s nutritional status and dietary habits can support data-driven decision making to optimize a patient-tailored treatment. There are a few nutrition-derived objective indicators that predict therapeutic outcomes and are used in clinical practice to adjust CID treatments. For instance, Crohn’s Diease patients experiencing extended nutritional deprivation are at increased risk of refeeding syndrome; European Society for Clinical Nutrition and Metabolism (ESPEN) guidelines recommend nutritional supplementation of phosphate and thiamine in such cases (26). In addition, it is well-documented that malnutrition promotes increased risk of morbidity and mortality following surgery in IBD patients (31, 32). Hence, if malnourishment is documented, nutritional support following emergency surgery is commonly recommended (26).

One ambitious goal of precision medicine is to emhance the amount and accuracy of data that describe a patient’s nutritional status, in order to identify novel biomarkers that can predict the clinical outcome of possible treatments. To achieve this goal, nutritional status data should be analyzed in close combination with other personal data as the effects of nutrients are moderated by other influencing factors such as host genetics, body composition or the intestinal microbiome (33). In a seminal study by Zeevi et al. (34), the authors collected longitudinal data from 800 participants including blood glucose levels, microbiome structure and function, nutritional status, and dietary behavior. On the basis of these data, a machine-learning algorithm was devised that was able to predict individual postprandial glycaemic response to a given meal Zeevi et al. (34). This example illustrates that the potential of nutritional information for identifying biomarkers for individual metabolic responses can be substantially potentiated when coupled with additional personal data, such as that related to the intestinal microbiome. Although the construction of such mathematical predictors relies on large cohorts and longitudinal data acquisition, similar approaches may also be applied to clinical CID patient cohorts, where nutritional data is incorporated in the predictor. Such predictors could assists clinicians in making personalized treatment decisions.

Moreover, the possibility of deriving prescriptive biomarkers from nutritional data will further increase as more molecular mechanisms describing the interplay among immunological pathways, the microbiome, and nutrients are elucidated. A series of studies within the past decade provided new evidence that the microbiome could act as a crucial intermediary between diet and inflammatory diseases (35). This complex relationship between dietary compounds, the microbiome, and inflammation is probably best elucidated for the anti-inflammatory effects of microbiome-derived butyrate. Butyrate and other short-chain fatty acids (SCFAs) are produced in large amounts through fermentation of dietary fibres by certain bacterial species (e.g. *Bacteroides fragilis*) that possess the enzymatic machinery to degrade those compounds (36). The diet-dependent and immunomodulating role of butyrate is especially interesting in the light of recent studies that demonstrated an association between colonic butyrate with clinical outcomes of CID therapies. For instance, it has been shown that butyrate levels are associated with clinical remission following anti-TNFα therapy in IBD patients (10, 37). Similarly, the efficacy of anti-CTLA-4 immunotherapy has been reported to be associated with the proportion of specific butyrate-producing bacteria within melanoma patients’ gut microbiota (38, 39). Moreover, a reduction in colonic butyrate-producing bacteria has been reported in HIV infection (40) and appears to affect the response to antiretroviral therapy (41). Such studies emphasise the potential to combine dietary information and microbiome data to approximate intestinal butyrate production capacity and, hence, individual responses to therapies.

Several molecular mechanisms have been elucidated how butyrate interacts with immunological pathways, illustrating the compound’s central role in CIDs. Smith et al. (14) have identified specialized butyrate-sensing receptors expressed by anti-inflammatory regulatory T-cells, whose differentiation is stimulated by butyrate (42). Butyrate also functions as a potent inhibitor of histone deacetylase enzymes, thereby linking microbial metabolites to the regulation of host transcriptional profiles (43, 44). Furthermore, Li et al. (45) have shown in a cell culture model that butyrate activates pyruvate kinase M2, leading to substantially altered cell metabolism, and thereby suppressing the proliferation of colorectal cancer cells.

Besides butyrate and other SCFAs, gastrointestinal microorganisms transform dietary components to contribute a wide range of additional compounds to the human metabolome (46). Future research on the role of these metabolites in health and disease will yield additional biomarkers and targets for personalized treatment of CIDs.

## Personalized Nutritional Interventions

Nutrition is likely the largest toolbox we have at hand to influence both metabolic processes in the human body and the intestinal microbiome’s structure and function. Thus, nutritional interventions represent a promising strategy for personalizing CID treatments. Nevertheless, various generalized nutritional recommendations, established over the past four decades in the context of chronic diseases, have not noticeably diminished their incidence (47).

New targets for nutritional interventions are expected to emerge as our mechanistic understanding of inflammatory diseases and the effect of nutrition on the immune system expands. For example, the above-mentioned anti-inflammatory effect of butyrate and its involvement in maintaining gastrointestinal health has promted researchers to explore using targeted dietary interventions to increase its intestinal production. Marino et al. (35) have shown that administration of a diet yielding high butyrate levels through gut microbial fermentation enhanced gut integrity, increased the number and activity of regulatory T cells, and decelerated the progression of diabetes in a diabetic mouse strain model. Such results exemplify the potential of dietary interventions to specifically target immunomodulating microorganisms. Thus, quantification of patient-specific activity of molecular processes (e.g. *via* coupled metagenomics and metabolomics) associated with the maintenance or initiation of CID remission are highly promising indicators for the efficacy of nutritional interventions targeted to modulate them.

Another intriguing development with implications for nutritional interventions are recent findings that the therapeutic effects of drugs are mediated through complex interactions between the bioactive agent, dietary compounds, and microorganisms (48, 49). These include pharmaceuticals frequently administered to treat certain CIDs, such as sulfasalazine or metformin (49, 50). In the case of metformin, a medication for the treatment of type 2 diabetes, Pryor et al. (50) have shown using a *Caenorhabditis elegans* model system that nutrition influences the animal’s response to the drug, and that the effects are mediated by the gut bacterium *Escherichia coli*. Specifically, metformin’s impact on host metabolism and lifespan are attributed to an increased production of agmatine, which further depends on nitrogen-containing compounds in the nematode’s diet, namely amino acids, amino sugars, and nucleotides. As part of the same study, reanalysis of microbiome data from four independent human cohorts indicated that the abundance of bacteria capable of producing agmatine increased in conjunction with metformin treatment. This suggests that similar synergistic interactions between the drug and nutrients may occur also in humans. In general, a mechanistic understanding of nutrient-drug interactions could pave the way to personalized CID treatment strategies that combine pharmacological and nutritional interventions.

Nutritional interventions that specifically target colonic microorganisms might be hindered by nutrient absorption in the small intestine. For instance, the vitamin niacin has been shown to beneficially affect intestinal homeostasis and decrease susceptibility to intestinal inflammation in a mouse model system (51). Fangmann et al. (52) employed a food-technological approach to deliver high amounts of nicotinic acid into the colon by micro-encapsulating the compound, thereby delaying its release until the capsules reached the ileocolonic region. *In vivo* administration of the capsules in humans changed the microbiota composition in ways that are commonly considered favourable; i.e. the increased abundance of *Bacteroidetes*. In addition, biomarkers of systemic insulin sensitivity and metabolic inflammation improved without observable negative side-effects (e.g. facial flushing) that have been described for un-capsulated orally administered niacin (52). Thus, food-technological approaches may promote the development of nutritional interventions for precision medicine by increasing the intervention efficacy and reducing unwanted side effects. In addition, several preclinical models demonstrated the potential of microbiome-directed nutritional interventions for the treatment of malnutrition and its associated inflammatory complications (53–55) and promising initial results were recently gained in preliminary clinical studies (56, 57).

## Challenges and Potential Solutions

### Precision in Dietary Assessment

Technological advances over the past decade have elevated the degree of precision with which a person can be characterized on both the genetic level (i.e. genomics) and phenotypic level (i.e. transcriptomics, proteomics, metabolomics). Available methods for assessing the environmental factor of *nutrition* (in terms of the person’s food consumption and habits) do not currently provide the same degree of detail in most cases. An exception is nutrition provided during intensive care, where nutritional intake is usually well documented, e.g. for preterm infants in neonatal intensive care units (58). In most other human cohort studies, nutrition is typically recorded using dietary questionnaires. Such questionnaires have the disadvantage that realiability of the data obtained may be limited, since it is based on the subjective perception of the study participant (59). Several software solutions (mainly mobile apps) have emerged that aim to increase dietary data quality (e.g. *via* incorporating automatic food item recognition from images), but that entail their own data acquisition shortcomings as reviewed elsewhere (60). While some of these solutions are already in use in biomedical research projects [e.g. (61)], a major issue remains: the dietary information obtained cannot be treated as objective and thereby does not meet the criteria for a source of potential medical biomarkers (62).

A promising approach to address this challenge is the identification of novel food intake biomarkers, which are molecularly-based objective indicators derived from human samples (63). The idea is to estimate previous dietary intake by measurements of dietary compounds or derived chemicals in human matrices such as blood, urine, faeces, hair, or dental calculus. Ongoing research focuses on the identification and evaluation of a wide range of different food intake biomarkers using metabolomics techniques (64). If proven applicable, such biomarkers will reveal new links between nutrition and inflammatory disease mechanisms.

### Nutrition and the Curse of Dimensionality

Food is molecularly complex. Online Databases such as FooDB (65) or FoodData (66) enable users to approximate the amount of macro- and micronutrients in a given diet. Thus, if personal dietary information is available for a large study cohort involving CID patients, one could statistically test for associations between individual nutrients and the patients’ disease manifestation and progression parameters. Yet, as Bauer et al. (27) pointed out, this approach would be hindered by the so-called *curse of dimensionality*, where the number of features (nutrients) quickly becomes larger than the number of samples (individuals), which often makes it difficult to distinguish real differences from differences that occur by chance. The true integration of omics data has been a major task in biomedical research in recent years, and several promising bioinformatics approaches have emerged [see Pinu et al. (67); Huang et al. (68) for reviews].

The basic idea behind multi-omics data analysis is to combine multiple biological features in a single analysis, in order to simulate phenotypic and environmental complexity and interrelatedness of biological systems as close as possible to the true nature of things. According to de Toro-Martín et al. (69), this includes deep phenotyping, physical activity, food behavior, and dietary habits in combination with multi-omics data. In fact, multi-omics data analysis might be a major driving force on the way to personalized medicine; some progress has been made in cancer research in particular (70), though to date none of these tools has enough predictive power for routine clinical use. This might be, because many tools still apply a sequential approach by analyzing one data layer after another and integrating the results post-analysis. As de Anda-Jáuregui and Hernández-Lemus (71) pointed out, biological processes and phenomena are not comprised of single, independent layers of biological features, and therefore algorithms that can simultaneously analyze multiple data types are preferred.

In multi-omics data analysis, an important distinction must be made between candidate/hypothesis-driven methods and more exploratory approaches employing dimensionality reduction techniques, such as principal component analysis. While the former has a potential drawback of information loss if the full data collected are no incorporated, the latter shows weaknesses in integrating biological system background knowledge. In addition, integration of non-omics data, like clinical phenotypes and nutritional data with omics data layers (e.g. gene variants, transcriptomes and microbiome data) is even more challenging, due to the heterogeneity of data types, possible interactions, and the existence of sub-phenotypes (72). To address those points, we need complex mathematical and bioinformatics methods [see Bersanelli et al. (73)] for a review] and careful attention to the preparation (such as standardization and normalization) and quality control in the different data types. Tools evolving from the field of systems biology, like metabolic network and pathway analysis, are incorporating known interactions between genes, proteins, micronutritional supply, and molecules, and can contribute significantly to the rapidly developing field of multi-omics analysis (74).

### Identifying Molecular Mechanisms

A major challenge in the identification of potential nutritional interventions is the elucidation of the molecular mechanisms illustrating how nutrition and specific dietary compounds influence immunological pathways. High-throughput technologies such as metagenomics and metabolomics have been applied to describe quantitative associations of personal nutritional data with metabolism, inflammation, and the microbiome in health and disease (75–77). Yet, these associations usually do not allow conclusions on underlying molecular mechanisms.

To investigate mechanisms, experimental model systems can be used, which provide valuable insights into key aspects of the molecular links between nutrition, immune system response, microbial processes, and inflammation (78). Animal models (e.g. mice, *C. elegans*) or cultures of established cell lines can be applied to elucidate general mechanisms and to screen many different combinations of potential influencing factors (48, 50). However, the translation of such results to human subjects is limited and cannot represent the heterogeneity in immune responses among human individuals. A promising alternative are *ex vivo* and *in vitro* model systems where biomaterial (e.g. tissue, cells, stool) are directly sampled from human individuals. *Ex vivo* and *in vitro* model systems enable large-scale phenotyping experiments under controlled conditions, and make it possible to link results directly with the individuals’ unique characteristics. Thus, such model systems are of vast interest in precision medicine. Available and predicted future model systems for human immunology are reviewed in detail Wagar et al. (78).

Computer models of biochemical processes are powerful tools for investigating the effect of nutrition on human metabolism and gut microbial processes. Various modelling methods exists [see Kumar et al. (79) for review], which share the common feature that system elements, namely metabolites, proteins, and genes, are represented in nodes within a network, where edges represent known relationships such as biochemical transformations, gene expression, and regulation. Thus, these network models allow predictions about metabolic flux distributions through metabolic networks in a given nutritional environment including the role of individual genes and proteins (79). The *in silico* simulations are vastly scalable, which enables researchers to perform simulations for a wide range of different scenarios (e.g. diets) as well as potential perturbations. Furthermore, theoretical models can be parametrized based on different data (e.g. abundance of specific proteins, transcripts, microorganisms, diet) from human individuals in order to frame the model to represent the individual’s conditions (50, 74). Results obtained from *in silico* models are useful to generate hypotheses about complex molecular mechanisms, which can subsequently be scrutinized by targeted experiments.

## Discussion

Clinicians have always striven to provide the best recommendations based on the patient’s characteristics and particular disease manifestation (23). Since nutrition is an important factor with vast impact on human health, nutritional interventions are often considered promising components in the treatment of a wide range of diseases. In some diseases, for which the molecular pathophysiology is well-understood, nutritional interventions have been proven to be highly effective, for instance in the treatment of phenylketonuria or coeliac disease (80). It is the ambitious goal of precision nutrition in chronic inflammation to achieve similar success with the help of nutrition-derived biomarkers and personalized nutritional interventions. This is a difficult task since CIDs arise from complex gene-microbiome-environment interactions (2) in which most underlying molecular mechanisms remain obscure. In this review, we discussed recent studies which address this issue and revealed nutrition’s vast and unexhausted potential in the treatment of CIDs by elucidating its impact on disease-related molecular pathways. Based on the applied methodologies in the reviewed studies and the current challenges discussed, we emphasize that future research in the field of nutrition in precision medicine for CIDs should focus on: (i) obtaining detailed nutritional data alongside omics-data (i.e. genomics, metagenomics, metabolomics) in human cohort studies in clinical contexts as well for population-level cohorts; (ii) development of novel mathematical methods to integrate different data sources in a systems biology framework that represents the relationship between measured molecular features; and (iii) elucidating molecular mechanisms describing how nutrition affects immunological pathways, including the modulating effects of drugs and the intestinal microbiota.

## Author Contributions

ML, KSchw, and SW conceptualized the review manuscript. SW took lead in writing the manuscript with contributions by TD, KSchl, KSchw, CKn, and JZ. All authors contributed to the article and approved the submitted version.

## Funding

The authors acknowledge the support by the Cluster of Excellence 2167 - “Precision medicine in chronic inflammation” - Deutsche Forschungsgemeinschaft, the Collaborative Research Centre CRC1182 "Origin and Function of Metaorganisms", and the Research Unit FOR5042 "miTarget - The Microbiome as a Target in Inflammatory Bowel Diseases". The funders had no role in study design, data collection and analysis, decision to publish, or preparation of the manuscript. 

## Conflict of Interest

The authors declare that the research was conducted in the absence of any commercial or financial relationships that could be construed as a potential conflict of interest.
